# Replication capacity and adaptability of a severe fever with thrombocytopenia syndrome virus at different temperatures

**DOI:** 10.1371/journal.pone.0188462

**Published:** 2017-11-30

**Authors:** Yan Feng, Changping Xu, Cixiu Li, Junfen Lin, Zhongfa Wang, Yanjun Zhang, Jianmin Jiang, Yiyu Lu

**Affiliations:** 1 Key Laboratory of Emergency Detection for Public Health of Zhejiang province, Zhejiang Provincial Centre for Disease Control and Prevention, Hangzhou, Zhejiang province, China; 2 Zhoushan Entry-Exit Inspection and Quarantine Bureau, Zhoushan, Zhejiang province, China; University of Kansas Medical Center, UNITED STATES

## Abstract

Severe fever with thrombocytopenia syndrome (SFTS) is an emerging disease caused by the SFTS virus (SFTSV). Although fever and thrombocytopenia are the typical manifestations of SFTS, a specific SFTS case with no fever was observed in Zhejiang, China. In this report, we aimed to explore the probable reason for the absence of fever by analyzing the genetic characteristics and temperature sensitivity (ts) of the SFTSV strain ZJ2013-06, which was isolated from the specific case. Phylogenetically, different clusters of SFTSV strains circulated in Zhejiang. ZJ2013-06 was farthest from ZJ2014-02, an isolate belonging to a Chinese dominant cluster, and nearest to the coastal strain NB24/CHN/2013. Ts tests, performed on Vero cells at 37°C and 39°C, indicated that ZJ2013-06 had restricted replication at 39°C. Its viral loads were substantially reduced at 39°C compared with that at 37°C (approximately 100-fold reduction) and were significantly lower than that of ZJ2014-02 at 39°C (*P* < 0.01). By adaptive culture at 39°C, the induced strain ZJ2013-06-P7 was obtained. Owing to a reverse mutation (S1616), ZJ2013-06-P7 lost the ts of the original strain, displaying similar replication processes with NB24/CHN/2013. The results indicated that the amino acid residue 1616 was related to the ts characteristics of ZJ2013-06. Our study revealed that ZJ2013-06 was temperature-sensitive and may be related to the absence of fever in our case.

## Introduction

Severe fever with thrombocytopenia syndrome (SFTS) is an emerging tick-borne hemorrhagic fever caused by SFTS virus (SFTSV), a new member of the *Phlebovirus* genus in the family *Bunyaviridae*. SFTS was first discovered in rural areas of central China in 2009 [1], and has been reported or confirmed in at least 23 provinces of mainland China, South Korea, Japan, and the United States of America as of 2016 [2–5]. The major clinical manifestations of SFTS include acute fever (temperature of 38°C or more), gastrointestinal symptoms, myalgia, dizziness, gingival hemorrhage, conjunctival congestion, thrombocytopenia, and leukocytopenia. Patients with severe disease may progress to multi-organ dysfunction, diffuse intravascular coagulation (DIC), shock, and death [6, 7]. The case-fatality rate ranges from 12% to 30% in different areas of China [1, 8, 9] and has been reported to be as high as 31% in Japan [10] and 32.6% in South Korea [11]. Moreover, the disease is believed to be transmitted through tick bites and contact with infectious blood and secretion [12–14].

Fever and thrombocytopenia are the typical manifestations of SFTS. Retrospective studies have shown that the simple pooled positive rate of fever is higher than 94% [10, 15, 16]. However, a mild case reported in Japan [17] and a specific case diagnosed in Dianshan county of Zhejiang exhibited no fever during the whole admission period [18]. The patient in Zhejiang province presented symptoms of nausea, retching, and anorexia; moreover, laboratory results indicated that thromobocytopenia, leukopenia, and liver and brain damage occurred [18]. Although most of these symptoms suggested that the patient was infected with SFTSV, and SFTSV infection was ultimately confirmed by reverse transcription polymerase chain reaction (RT-PCR), the patient did not experience any fever during the entire hospitalization period. Ye et al. indicated that a low exposure dose of SFTSV may result in the absence of fever [18]. This explanation could also support the occurrence of a mild clinical course of a case in Japan [17]. Moreover, the genetic diversity of SFTSV ZJ2013-06, a strain isolated from the specific case, may also contribute to the absence of fever through reducing viral pathogenicity [18]; however, no evidence or data have supported this conclusion.

Accordingly, in this study, we evaluated the genetic characteristics, replication capacity, and adaptability of the isolate ZJ2013-06 at different temperatures, with the goal of identifying the biological features of the strain that may explain the absence of fever in our specific case.

## Materials and methods

### Viruses

Five SFTSV strains, isolated from confirmed SFTS cases in Zhejiang province, China during 2012 and 2014, were used in this study. Of the five strains, ZJ2012-03, ZJ2013-07, ZJ2014-01, and ZJ2014-02 were first isolated by Zhoushan, Ningbo, Huzhou, and Taizhou Municipal CDCs, respectively, and then sent to Zhejiang provincial CDC. Isolation of the strains was used for routine diagnosis of the patients. From the perspective of severity of illness, patients infected with ZJ2013-07 and ZJ2014-01 strains were discharged, whereas patients infected with ZJ2012-03 and ZJ2014-02 died. Another strain, ZJ2013-06 (Genbank accession no. KP280206), also originated from Zhoushan city, an island in Zhejiang. The patient infected with ZJ2013-06 presented the clinical symptoms of severe leukopenia (1.3 × 10^9^/L), thrombocytopenia (74 × 10^9^/L), liver dysfunction (AST 469 U/L), and brain damage; meanwhile, she was treated with recombinant human granulocyte colony-stimulating factor (GM-CSF; 75 μg) and compound glycyrrhizin [18]. Because the patient presented no fever during the entire admission period, we defined her as a specific case [18].

### Viral RNA extraction and whole-genome sequencing

Genomic RNA from ZJ2012-03, ZJ2013-07, ZJ2014-01, and ZJ2014-02 was extracted from 200 μL culture fluid and eluted into 30 μL RNase-free water using an RNeasy Mini Kit (Qiagen, Hilden, Germany) according to the manufacturer’s instructions. The SFTSV genome was generated by RT-PCR using a PrimeScript One Step RT-PCR Kit (TaKaRa Biotechnology Co., Shiga, Japan) and primer sets published previously [19]. RT-PCR products were purified and sequenced by Sangon Biotech Co., Ltd. (Shanghai, China), with the primer sets used in amplification. The whole genome sequences of the Zhejiang strains ZJ2012-03, ZJ2013-07, ZJ2014-01, and ZJ2014-02 were submitted to GenBank with accession numbers KY273264-KY273267 for the L gene, KY273268-KY273271 for the M gene, and KY273272-KY273275 for the S gene.

### Phylogenic analysis

Complete L gene sequences representative of the SFTSV diversities were downloaded from NCBI and aligned with sequences generated in this study using MAFFT version 7 with the E-INS-i algorithm. The final alignment lengths were 6255 bp. Phylogenetic trees were inferred from 40 strains using the maximum likelihood (ML) method available in PhyML version 3, with the GTR + Γ nucleotide substitution model and a subtree pruning and regrafting (SPR) topology searching algorithm. Of the 40 representative strains used in phylogeny analysis, 18 were isolated in Zhejiang province, China during 2011 and 2013, and 22 strains were from other provinces of China, Japan, and South Korea.

### Replication capacity of ZJ2013-06 and ZJ2014-02 at 37°C and 39°C

The ts of ZJ2013-06 and ZJ2014-02 was assessed by measuring the replication capacities of the two viruses in Vero cells at 37°C and 39°C, based on previous studies [20–22]. The Vero cell line was donated by the Chinese National Center for Disease Control and Prevention and cultured in growth medium (GM) containing 10% fetal bovine serum (FBS), 50 μg/mL gentamicin, and an additional 200 mM l-glutamine. The cells were adjusted to a density of 5 × 10^5^ cells/mL and then incubated at 37°C. After the monolayer formed, culture fluids of the two strains were diluted and titrated to the same concentration (6 × 10^4^ copies/mL), and 100 µL of each diluted preparation was then inoculated into fresh Vero cell monolayers in cell tubes. A total of 30 tubes were inoculated for each virus since three replicates were required for each temperature and each harvested time point. After inoculation, 15 tubes of each virus were incubated at 37°C and another 15 tubes were incubated at 39°C. Three tubes of each virus were harvested on the day of inoculation and on days 3, 5, 7, and 9 after infection. After two freeze-thaw cycles, all harvests were detected using quantitative real-time RT-PCR (qRT-PCR) [23]. Proliferation curves were plotted using the common logarithm of copy number and the time of inoculation.

### qRT-PCR

The qRT-PCR assay developed previously was performed using a One Step PrimeScript RT-PCR (Perfect Real-time) Kit (TaKaRa Biotechnology Co.) and a primer-probe set designed to detect the M gene of SFTSV [23]. Each reaction contained 12.5 μL of 2 × One Step RT-PCR buffer, 0.5 μL TaKaRa Ex Taq™ HS, 0.5 μL PrimeScript RT Enzyme Mix, 0.48 μM of each primer, 0.24 μM of probe, 5 μL ddH2O, and 5 μL RNA template in a final volume of 25 μL.

RNA standards were generated from the ZJ2014-02 strain. The M gene sequence (nt 907–1583) of ZJ2014-02 was amplified with primers that included the T7 promoter sequence. The amplified sequence was transcribed in vitro with T7 RNA polymerase (TaKaRa Biotechnology Co.) according to the manufacturer’s instructions. The synthetic RNA transcript from the M gene of SFTSV was purified, quantified, and then serially diluted to 10^1^ to 10^8^ copies/μL. The diluted RNA transcripts were detected as standards, and the standard curve is shown as the copy number plotted against the cycle number. Consequently, the linear regression equation obtained was y = -3.755x + 38.514, and the linear correlation between the common logarithm of copy number and Ct value was strong (r^2^ = 0.996).

### Amino acid sequence comparison

In order to search for amino acids specific to ZJ2013-06, the L gene sequences of 217 representative SFTSV strains were obtained from GenBank and compared with that of ZJ2013-06. Of the strains, 34 were isolated in Zhejiang, and the other 183 were isolated from other regions throughout the world.

### Adaptive culture of ZJ2013-06

To analyze the relationships between specific amino acids and the ts, ZJ2013-06 was continuously passaged in Vero cells at 39°C. Briefly, Vero cells were seeded at a concentration of 5 × 10^5^ cells/mL in cell tubes and incubated at 37°C. After monolayer formation, 100 μL culture fluid of ZJ2013-06 was inoculated, and the cell tube was incubated at 39°C for 7 days. The tubes were then frozen at -80°C for 2 h. The culture fluid was mixed thoroughly and harvested as the first passage of ZJ2013-06 (ZJ2013-06-P1). Next, 100 μL of culture fluid of ZJ2013-06-P1 was used for seven further passages (ZJ2013-06-P7), as per the method described for the first passage. The genome sequence of ZJ2013-06-P7 was generated and compared with the sequences of ZJ2013-06. Moreover, the replication capacity of ZJ2013-06-P7 was also evaluated and compared with that of NB24/CHN/2013, as described previously.

### Statistical analysis

Student’s t-tests were applied to compare the mean of copy numbers of ZJ2013-06 and ZJ2014-02 at different temperatures. One-way analysis of variance was used to test the differences in three dynamic changes on copy numbers among the different groups, and multiple comparison tests were conducted by Bonferroni correction. All statistical tests were two-sided and considered statistically significant at a *p* value of less than 0.05. Data entry and validation were carried out using EpiData (version 3.1), and data analysis was performed using SPSS for Windows (Version 22.0, Chicago, IL, USA).

## Results

### Phylogenetic analysis of ZJ2013-06

**Fig 1:**
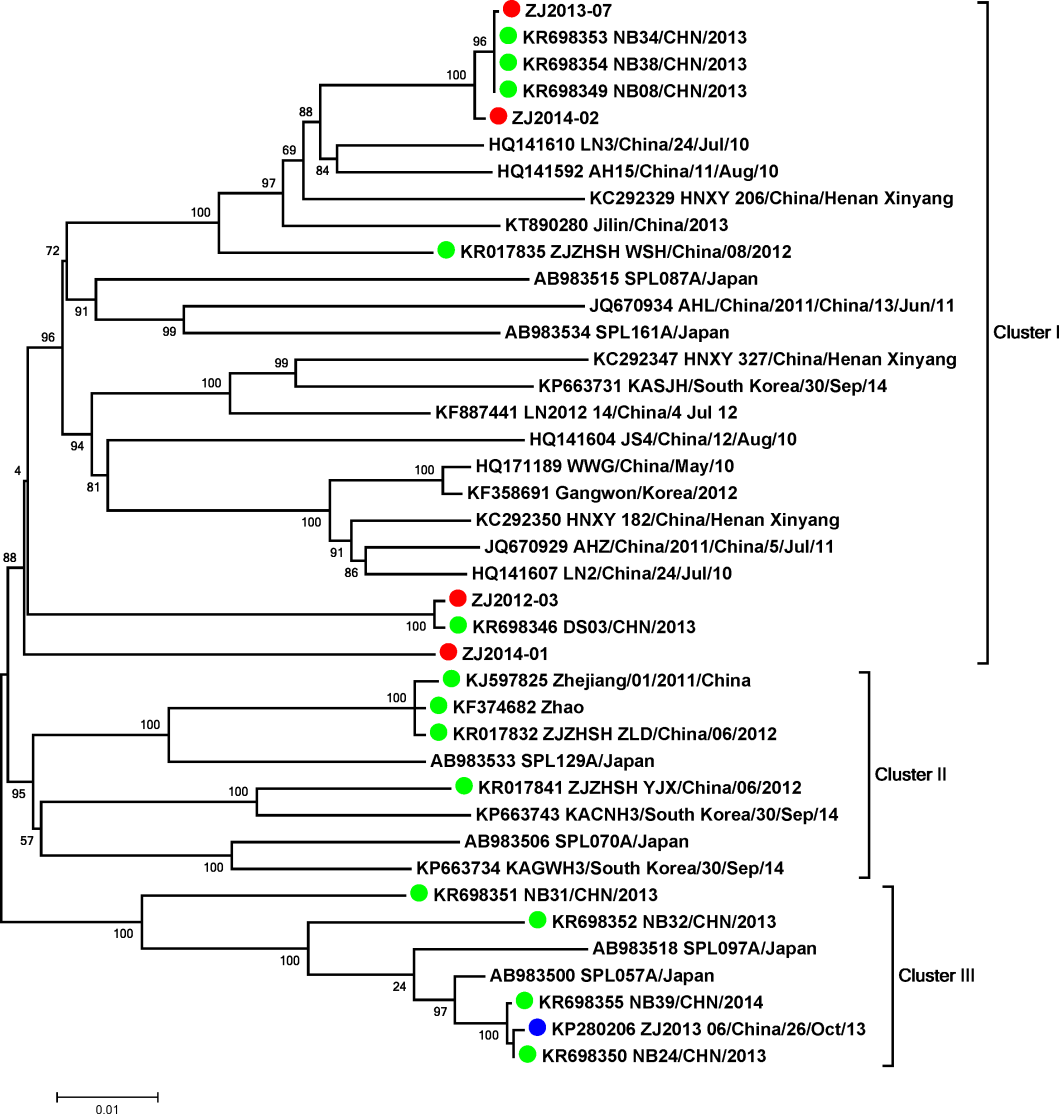
Phylogenic analysis of the whole sequence of the L gene of ZJ2013-06 and other 39 representative SFTSVs. The maximum likelihood (ML) tree was generated using PhyML version 3, with the GTR + Γ nucleotide substitution model and a Subtree Pruning and Regrafting (SPR) topology searching algorithm. SFTSV was divided into three clusters, labeled I, II, and III. Strains marked with red circles represent viruses isolated in Zhejiang and sequenced in this study. Strains marked with green circles represent viruses isolated in Zhejiang and sequenced previously. The strain marked with a blue circle is the ZJ2013-06 strain from the specific case with no fever.

Complete L gene sequences of ZJ2013-06 and 39 representative strains (17 from Zhejiang) were phylogenetically analyzed. Based on the phylogenetic tree (Fig 1), Zhejiang SFTSV strains were divided into three clusters. Of these clusters, cluster I contained eight Zhejiang strains (ZJ2013-07, ZJ2014-02, ZJ2012-3, ZJ2014-01, and four previously sequenced strains), and all strains originated from other provinces of China. Cluster II was formed by viral strains from Japan, South Korea, and Zhoushan Island of Zhejiang (published previously). The ZJ2013-06 strain, which was isolated from the specific SFTS case, was phylogenetically classified into cluster III with four Zhejiang strains and two Japanese strains. The four Zhejiang strain in cluster III all originated from Ningbo city, which is adjacent to where the ZJ2013-06 strain was isolated. Moreover, ZJ2013-06 had the closest relationship with NB24/CHN/2013 (a strain isolated from Ningbo city of Zhejiang in 2013), with six nucleotide differences on the L gene, and was farthest away from ZJ2013-07 and ZJ2014-02, with 276 and 275 nucleotide differences on the L gene. Phylogenetic tree (S1 Fig) constructed based on the S gene sequences showed the similar results with the L gene tree.

### The ts of the ZJ2013-06 strain

**Fig 2:**
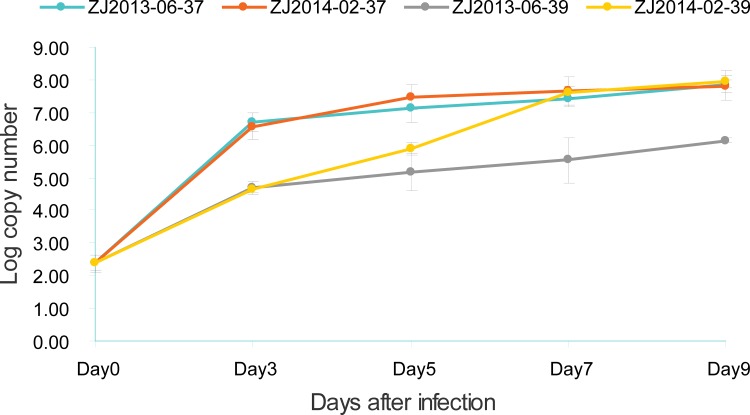
Proliferation curves of ZJ2013-06 and ZJ2014-02 in Vero cells at different temperatures.

In order to determine the ts of ZJ2013-06, the replication capacity of ZJ2013-06 was tested and compared with that of a typical strain, ZJ2014-02. The two strains were initially titrated to the same concentration (roughly 280 copies/reaction) and then cultured at 37°C or 39°C for 9 days. The replication capacities were determined by testing the viral loads of the two strains every other day (Table 1), and the dynamic changes are shown in Fig 2.

**Table 1:** Replication capacities of ZJ2013-06 at different temperatures.

Time	37°C (Mean ± SD)		*P* value	39°C (Mean ± SD)		*P* value
	ZJ2013-06	ZJ2014-02		ZJ2013-06	ZJ2014-02	
Day 0	280.50 ± 166.81	283.35 ± 148.81	0.90	280.50 ± 166.81	283.35 ± 148.81	0.90
Day 3	6.06 ± 4.36 × 10^7^	5.80 ± 4.88 × 10^7^	0.67	5.10 ± 1.91 × 10^5^	4.41 ± 0.81 × 10^5^	0.74
Day 5	1.65 ± 1.19 × 10^8^	3.50 ± 2.32 × 10^8^	0.34	2.35 ± 2.06 × 10^6^	7.84 ± 3.34 × 10^6^	0.12
Day 7	2.79 ± 1.42 × 10^8^	5.39 ± 4.78 × 10^8^	0.43	6.11 ± 4.91 × 10^6^	4.48 ± 1.75 × 10^8^	< 0.01
Day 9	9.50 ± 8.67 × 10^8^	6.62 ± 2.35 × 10^8^	0.93	1.40 ± 0.19 × 10^7^	9.29 ± 4.43 × 10^8^	< 0.01

At 37°C, ZJ2013-06 and ZJ2014-02 replicated well over time. Viral loads increased dramatically to 6.06 ± 4.36 × 10^7^ copies/reaction for ZJ2013-06 and 5.80 ± 4.88 × 10^7^ copies/reaction for ZJ2014-02 by day 3 and to 9.50 ± 8.67 × 10^8^ copies/reaction for ZJ2013-06 and 6.62 ± 2.35 × 10^8^ copies/reaction for ZJ2014-02 by day 9 after infection (Table 1). No significant differences were detected in copy numbers between the two strains. Analysis of the proliferation curve (Fig 2) also demonstrated that the two strains had roughly the same proliferation process, with the exception of a slight difference on day 5 after infection.

At 39°C, the replication of ZJ 2013–06 was limited. The viral loads of ZJ2013-06 decreased approximately 119-, 70-, 46-, and 68-fold on days 3, 5, 7, and 9 after infection, compared with that at 37°C (Table 1). However, the replication of ZJ2014-02 was not affected at 39°C. From the proliferation curve (Fig 2), branching first occurred on day 5 and then gradually increased. The most significant difference was detected on day 7 after infection (*P* < 0.01), with 6.11 ± 4.91 × 10^6^ copies/reaction for ZJ2013-06 and 4.48 ± 1.75 × 10^8^ copies/reaction for ZJ2014-02. On day 9 after infection, the viral load of ZJ2013-06 reached 1.40 ± 0.19 × 10^7^ copies/reaction, which was still markedly lower than that of ZJ2014-02 (9.29 ± 4.43 × 10^8^ copies/reaction; *P* < 0.01).

### Specific amino acids of the ZJ2013-06 strain

Amino acid alignments were performed for L gene sequences between ZJ2013-06 and the representative strains isolated from Zhejiang and other regions; the results are shown in Table 2. Based on the alignments with 213 strains (183 from other regions and 30 from Zhejiang), six amino acid sites were specific to ZJ2013-06, including S317, T343, V570, S1208, R1577, and N1616. With regard to the four Zhejiang strains in cluster III, ZJ2013-06 possessed five stable variations (S317, T343, S1208, R1577, and N1616) compared with NB31/CHN/2013, but possessed only one site (N1616) different from NB24/CHN/2013, NB32/CHN/2013, and NB39/CHN/2013. In addition, no stable variations were found in the amino acid sequences of the M and S gene of ZJ 2013–06.

**Table 2:** Specific amino acid sites possessed by the ZJ2013-06 strain.

Group/strains		No.	Amino acid sites					
		of strains	317	343	570	1208	1577	1616
ZJ2013-06		1	S	T	V	S	R	N
ZJ2013-06-P7		1	·^a^	·	·	·	·	S
ZJ strain in cluster 3	NB24/CHN/2013	1	·	·	·	·	·	S
	NB32/CHN/2013	1	·	·	·	·	·	S
	NB39/CHN/2013	1	·	·	·	·	·	S
	NB31/CHN/2013	1	A	S	·	T	K	S
Other ZJ strain		30	A	S	I	T	K	S
Other region strains		183	A	S	I	T	K	S

^a^‘·’represents the amino acid in that site is identical to the amino acid on the same site of ZJ2013-06

### Characteristics of the induced strain ZJ2013-06-P7

To further elucidate the ts of ZJ2013-06, the strain was adaptively cultured at 39°C, and the induced strain ZJ2013-06-P7 was obtained after seven-run passages. The genetic characteristics and ts of the induced strain were analyzed.

Amino acid sequence alignments showed there were no differences between ZJ2013-06-P7 and the original strain ZJ2013-06 on the M and S genes (results not shown), but one mutation was observed on the L gene of ZJ2013-06-P7. Amino acid residue 1616 was asparagine for ZJ2013-06, but was changed to serine for the induced strain ZJ2013-06-P7 (Table 3). This mutation made ZJ2013-6-P7 have the same amino acid at residue 1616 as all typical strains. Furthermore, owing to this reverse mutation, ZJ2013-06-P7 was genetically identical as NB24/CHN/2013 (a typical strain having a close relationship with the original strain ZJ2013-06 in the phylogenetic tree).

**Table 3:** Temperature sensitivity of NB24/CHN/2013 and ZJ2013-06-P7.

Time	NB24/CHN/2013 (Mean ± SD)		*P* value	ZJ2013-06-P7 (Mean ± SD)		*P* value
	37°C	39°C		37°C	39°C	
Day 0	10.49 ± 3.90	9.59 ± 3.27	0.77	10.91 ± 1.82	13.85 ± 3.86	0.30
Day 3	1.10 ± 0.59 × 10^4^	0.45 ± 0.29 × 10^4^	0.16	1.48 ± 1.16 × 10^4^	0.52 ± 0.10 × 10^4^	0.22
Day 5	2.38 ± 1.53 × 10^5^	0.26 ± 0.07 × 10^5^	0.19	2.25 ± 1.91 × 10^5^	1.24 ± 0.79 × 10^5^	0.44
Day 7	1.40 ± 0.53 × 10^6^	0.98 ± 0.37 × 10^6^	0.39	3.82 ± 1.88 × 10^6^	1.75 ± 0.63 × 10^6^	0.15
Day 9	1.33 ± 0.39 × 10^7^	1.50 ± 0.33 × 10^7^	0.50	9.53 ± 4.10 × 10^6^	7.92 ± 5.11 × 10^6^	0.69

**Fig 3:**
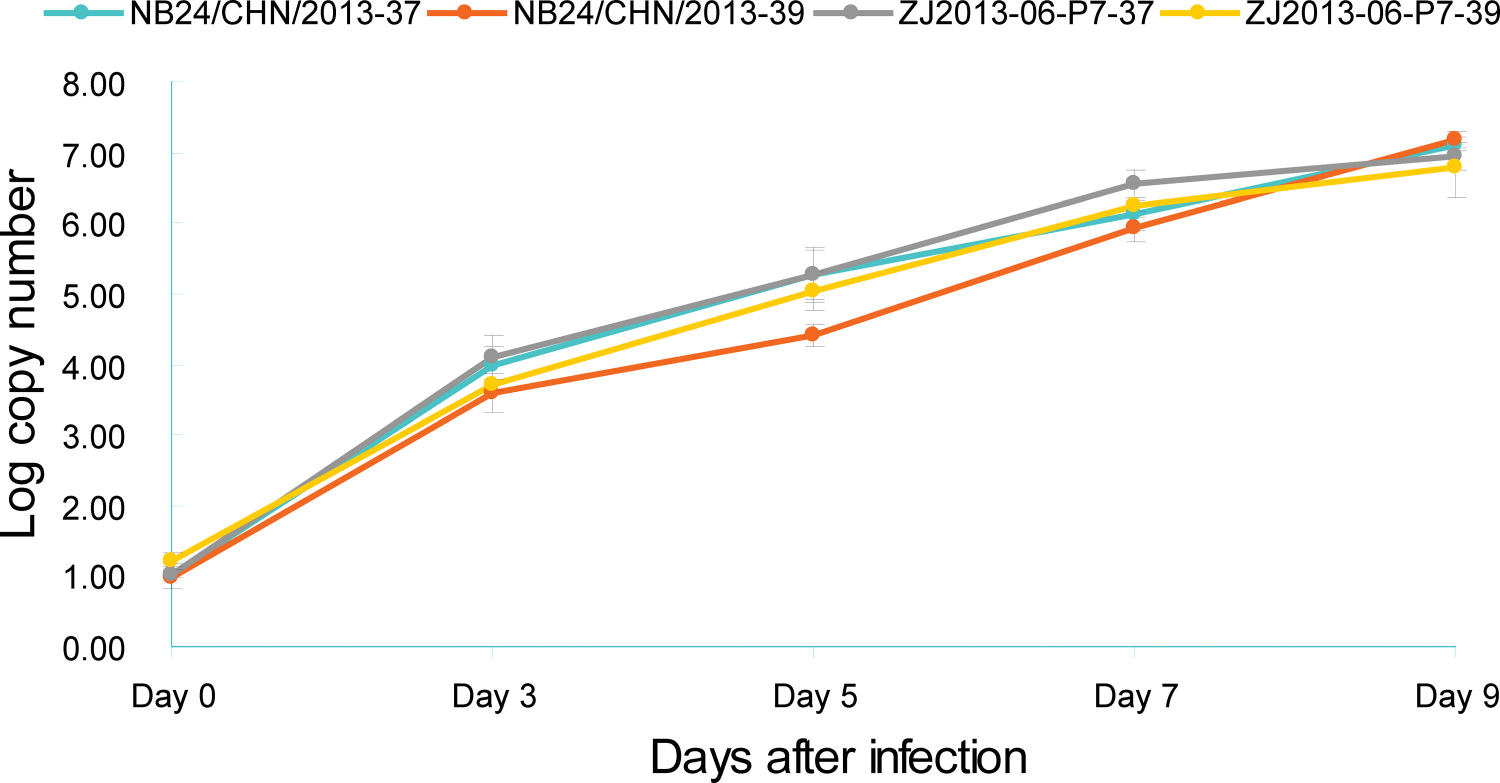
Proliferation curves of ZJ2013-06-P7 and NB24/CHN/2013 in Vero cells at 37°C and 39°C.

The ts of ZJ2013-06-P7 was determined and compared with that of NB24/CHN/2013. The original concentrations of the two strains were titrated at roughly 10 copies/reaction on inoculation day. Analysis of dynamic changes in viral loads (Table 3) showed that ZJ2013-06-P7 replicated well at both 37°C and 39°C. Even the viral load tested at 39°C was slightly lower than that tested at 37°C at each time point (from 0.52 ± 0.10 × 10^4^ copies/reaction on day 3 to 7.92 ± 5.11 × 10^6^ copies/reaction on day 9 at 39°C; from 1.48 ± 1.16 × 10^4^ copies/reaction on day 3 to 9.53 ± 4.10 × 10^6^ on day 9 at 37°C); however, no significant difference was observed (*P* > 0.05). In the proliferation curve (Fig 3), the proliferation processes of ZJ2013-06-P7 showed good consistency at the two temperatures.

In order to control the experiment and clarify the relationship among the mutation, ts, and the absence of fever, the replication capacity of NB24/CHN/2013 was also tested at the same time. The replication process of NB24/CHN/2013 was similar to that of ZJ2013-06-P7 (Fig 3). The differences in viral load tested between 37°C and 39°C were not significant (*P* > 0.05; Table 3), and the proliferation of NB24/CHN/2013 was not restricted at 39°C.

## Discussion

In this study, we performed phylogenetic analysis of different clusters of SFTSV strains that had cocirculated in Zhejiang. Generally, strains (clusters II and III) from the coastal areas of Zhejiang (Zhoushan Archipelago Island and Ningbo city) were found to be related molecularly to Japanese or Korean strains, consistent with the conclusions of previous studies [24, 25]. Our results also provided data to support that SFTSV infection in Japan or Jeju island of South Korea was closely related to Zhejiang strains [24]. Another type of Zhejiang strain (cluster I) belonged to a Chinese dominant cluster and was designated in the same branch as the SFTSV strains from central or northeastern China. Notably, the Zhejiang strains in cluster I contained viruses isolated from not only coastal but also inland areas. Hence, we inferred that different clusters of SFTSV had circulated in Zhoushan Island during the same time period.

Importantly, ts can result in limited or decreased viral replication at a restrictive temperature. In our study, ZJ2013-06 displayed restricted replication at 39°C (the viral loads of ZJ2013-06 were reduced by approximately 100-fold compared with that at 37°C), indicating that ZJ2013-06 was a ts mutant of SFTSV. Previous studies have shown that ts may be associated with viral attenuation [21, 22, 26], i.e., attenuated viral strains may possess these characteristics; nevertheless, the clinical symptoms of the patient, including sever leucopenia, thrombocytopenia, and liver and brain damage, suggested that the temperature-sensitive strain ZJ2013-06 was a virulent strain.

Next, we examined how the replication characteristics of ZJ2013-06 affected the host, i.e., if the ts of ZJ2013-06 was related to the absence of fever in our specific case. To this end, the replication capacity of the typical strain ZJ2014-02 (belonging to the Chinese dominant lineage and having the greatest distance from ZJ2013-06 in the phylogenetic tree) was tested and compared with that of ZJ2013-06. Interestingly, ZJ2014-02 replicated well at 39°C, and the viral load of ZJ2014-02 was significantly higher than that of ZJ2013-06, showing no ts. In order to further verify the ts of ZJ2013-06, the replication capacities of another typical strain, NB24/CHN/2013 (the strain with the closest distance to ZJ2013-06), was also determined. Comparison of the viral loads tested at 37°C and 39°C confirmed that NB24/CHN/2013 had no ts as well. These results indicated that ZJ2013-06 had biological features different from those of typical SFTSV strains.

Based on sequence analysis, the ZJ2013-06 had a specific amino acid (N1616) compared with the sequences of SFTSV strains isolated worldwide (available on the NCBI website); hence, we inferred that the N1616S mutation may be related to the ts of ZJ2013-06. To verify this, the ZJ2013-06 strain was continuously passaged at 39°C. Based on the reverse mutation at position 1616, the adaptive strain ZJ2013-06-P7 lost the ts characteristics of the original strain, which demonstrated that the amino acid at residue 1616 may play a key role in the ts of ZJ2013-06. Moreover, ZJ2013-06-P7 is considered identical to NB24/CHN/24 genetically. Because the patient infected with NB24/CHN/2013 developed a fever (highest temperature of 38.8°C) throughout the course of the disease, we inferred that the ts of ZJ2013-06 may be the reason for the absence of fever.

A previous study in Zhejiang showed that 9% (11/120) of serum samples from local healthy individuals without symptoms were positive for antibodies to SFTSV [27]. Additionally, researchers in Japan reported an SFTS case with a mild clinical course and no fever [17]. Based on our results, there may be a few ZJ2013-06-like strains showing ts, which can result in atypical SFTSV infection.

Because only one SFTS case with no fever was reported in Zhejiang and sequences or isolates from other mild cases or cases with no fever were not available, we are not sure whether the ts characteristic is specific for the ZJ2013-06 strain or is universal for the SFTSVs isolated from other cases with no fever. Since different animals have varying body temperatures, it was not clear whether the ts characteristic of ZJ2013-06 was related to the host animal; further studies are required to evaluate this.

In conclusion, the ZJ2013-06 strain, isolated from a specific case with no fever, was a ts strain of SFTSV. Furthermore, ZJ2013-06-like strains may be circulating in nature, resulting in atypical SFTSV infections.

## Supporting information

S1 Fig Maximum likelihood tree constructed based on the S gene sequences of ZJ2013-06 and other 42 representative SFTSVs. The tree was generated using PhyML version 3, with the GTR + Γ nucleotide substitution model and a Subtree Pruning and Regrafting (SPR) topology searching algorithm. Strains marked with red circles represent viruses isolated in Zhejiang and sequenced in this study. Strains marked with green circles represent viruses isolated in Zhejiang and sequenced previously. The strain marked with a blue circle is the ZJ2013-06 strain from the specific case with no fever.
(TIFF)
