# Supplementary figures and images for: Replication capacity and adaptability of a severe fever with thrombocytopenia syndrome virus at different temperatures
Source: PLoS One. 2017 Nov 30;12(11):e0188462. doi: 10.1371/journal.pone.0188462 (PMC5708652; doi:10.1371/journal.pone.0188462)

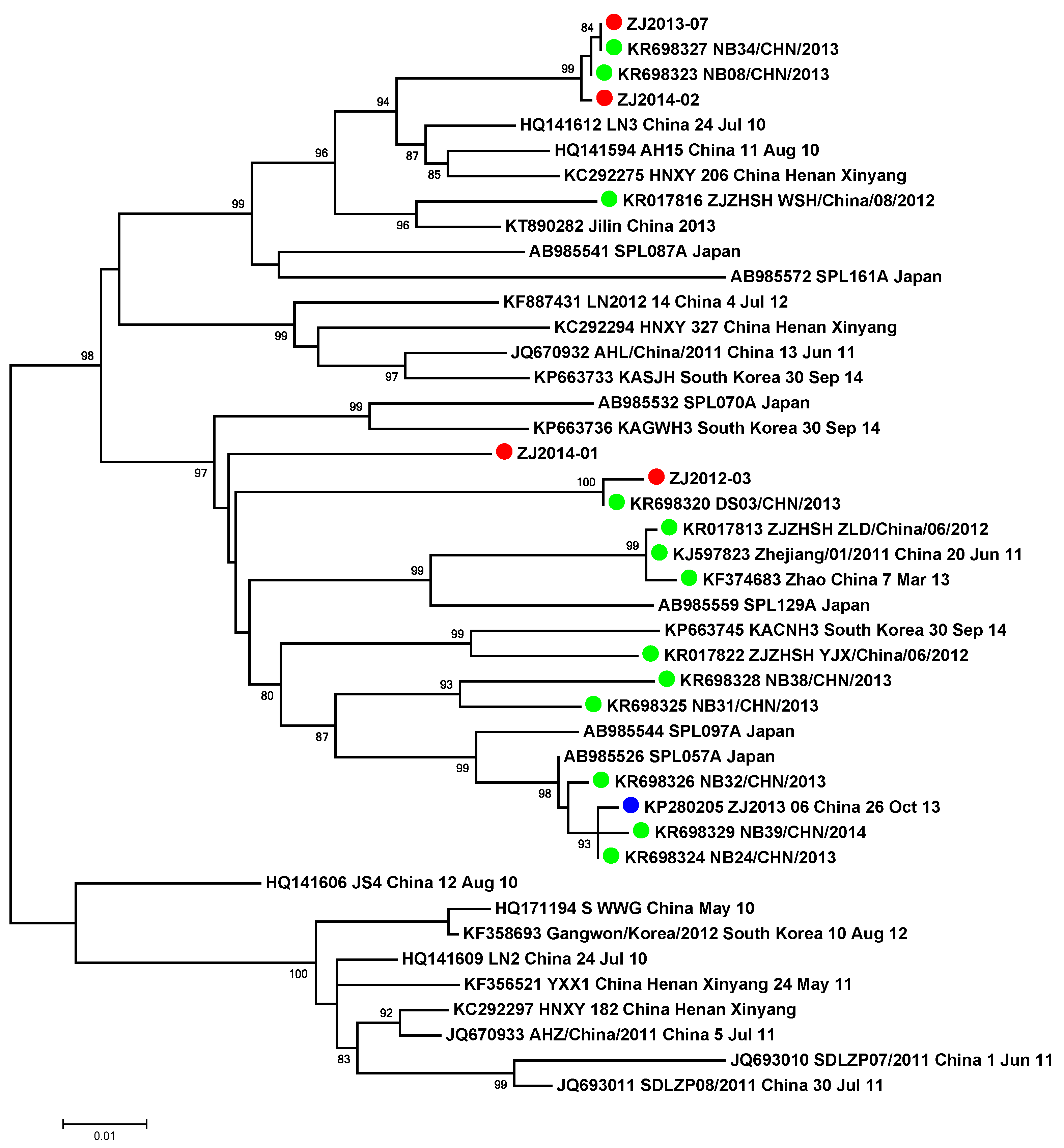

Supplement: S1 Fig — The tree was generated using PhyML version 3, with the GTR + Γ nucleotide substitution model and a Subtree Pruning and Regrafting (SPR) topology searching algorithm. Strains marked with red circles represent viruses isolated in Zhejiang and sequenced in this study. Strains marked with green circles represent viruses isolated in Zhejiang and sequenced previously. The strain marked with a blue circle is the ZJ2013-06 strain from the specific case with no fever. (TIFF) [file pone.0188462.s001.tiff]
